# Antinociceptive Action of *Moringa peregrina* is Mediated by an Interaction with α_2_-Adrenergic Receptor

**DOI:** 10.4274/balkanmedj.galenos.2020.2019.11.14

**Published:** 2020-06-01

**Authors:** Sahar M. Jaffal, Belal O. Al-Najjar, Manal A. Abbas, Sawsan A. Oran

**Affiliations:** 1 Department of Biological Sciences, Jordan University School of Science, Amman, Jordan; 2 Department of Pharmaceutical Sciences, Al-Ahliyya Amman University School of Pharmacy, Amman, Jordan; 3 Department of Medical Laboratory Sciences, School of Allied Medical Sciences, Al-Ahliyya Amman University, Amman, Jordan

**Keywords:** Adrenergic alpha-2 receptor antagonists, analgesics, molecular docking, Moringa peregrina

## Abstract

**Background:**

*Moringa peregrina (M. peregrina)* is an edible, drought-resistant tree that is native to semi-arid countries. It is used as a painkiller in folk medicine.

**Aims:**

To study the antinociceptive effects of the leaf extract of *M. peregrina* in mice.

**Study Design:**

Animal experimentation.

**Methods:**

We employed thermal (hot plate and tail-immersion tests) and chemical (writhing and formalin tests) pain models in male BALB/c mice (eight animals per group) to investigate the mechanisms involved in the antinociceptive actions of *M. peregrina*. Additionally, we identified the chemical constituents present in the extract of *M. peregrina* by using liquid chromatography-mass spectrometry analysis, and predicted the possible active constituents that interact with the receptor based on molecular docking simulations.

**Results:**

In the writhing test, 200 mg/kg of *M. peregrina* extract restricted abdominal cramps by up to 55.97% (p<0.001). Further, it reduced the time of paw-licking in the early and late phases of formalin test by up to 56.8% and 65.5%, respectively, as compared to the percentage inhibitions of 50.5% and 48.4% produced by 30 mg/kg diclofenac sodium in the early and late phases, respectively (p<0.05). This effect was abrogated by yohimbine (1 mg/kg, intraperitoneally), but not by methysergide (5 mg/kg, intraperitoneally), in the late phase only, which indicates that the action of *M. peregrina* in formalin test is not mediated by 5-HT2 serotonin receptors, but rather via α_2_-adrenergic receptors. In the hot plate test, but not on tail-immersion test, the high dose (400 mg/kg) of the extract increased the latency time after 30 minutes of its administration. Yohimbine antagonized the action of *M. peregrina* in the hot plate test. Based on LC-MS analysis, the major constituents found in *M. peregrina* methanolic extract were chrysoeriol 7-O-diglucoside, lupeol acetate, quercetin, and rutin. Depending on the molecular docking results, the activity of *M. peregrina* extract could be due to the binding of chrysoeriol 7-O-diglucoside, quercetin, and rutin to the α_2_-adrenergic receptor.

**Conclusion:**

Interaction with the α_2_-adrenergic receptor serves as a possible mechanism of the *M. peregrina* analgesic effect.

*Moringa *(Moringaceae) is a genus that comprises 13 species of trees and shrubs growing in the tropical and sub-tropical regions of our world (1). Various civilizations, such as Greek, Indian, and Egyptian, have utilized *Moringa* for many millennia. *Moringa* (mainly its leaves and fruits) has always been an integral part of people’s diet. Additionally, *Moringa* leaves were fed to the ancient Mauryan warriors of India to provide them with energy and relieve their pain (2).* Moringa peregrina *(*M. peregrina*) is a drought-resistant tree that grows in the dry or semi-arid countries located near the Red Sea such as Somalia, Syria, Palestine, and Yemen (3). The tree is known for its fast growth and can reach up to 3-10 meters in height after ten months of its cultivation (4). The leaves of *M. peregrina *are obovate, alternate, deciduous, and about 30-40 cm in length (2). Almost all parts of this plant are consumed by humans as a vegetable due to its taste, flavor, and nutritional value. The leaves of *M. peregrina* are great sources of proteins, vital elements (Ca^+2^, Mg^+2^, K^+1^ and Fe^+2^), essential amino acids, and vitamins such as vitamin A, C, and E (5,6).

*M. peregrina* is considerably used by people of various cultures in traditional healing practices during childbirth and for treating multiple disorders, such as malaria, fever, diabetes, abdominal pain, asthma, headache, constipation, muscle pain, hypertension, burns (7,8). Pharmacological studies have reported that *M. peregrina* has antimicrobial (9), *in vitro* antioxidant (8), anticancer (10), and antispasmodic properties (7) as well as immunomodulatory activities (both *ex vivo* and *in vivo*) (3). In rats, *M. peregrina* has demonstrated anti-inflammatory (11), antiulcer (12), antihyperglycemic (13), antihyperlipidemic (14), antihypertensive effects (15), and memory-enhancing activities (16).

Despite the use of *M. peregrina* in folk medicine as a painkiller (4), there is a paucity of detailed studies determining the analgesic effects of this plant. Previous work on *M. peregrina* includes only a preliminary investigation that employed writhing and hot plate tests (12). The purpose of this investigative study is to examine the effects of *M. peregrina* on the early and late phases of formalin test. Additionally, we performed tail-immersion test to study the effects of *M. peregrina* at the spinal level. Furthermore, we studied the mechanism by which *M. peregrina* exerts its action.

## MATERIALS AND METHODS

### Drugs

Diclofenac sodium was obtained from Novartis, *Switzerland*, whereas methysergide was obtained from Sigma-Aldrich, USA. Yohimbine was purchased from Tocris Bioscience (UK). All the drugs were freshly prepared in a sterile normal saline solution and administered intraperitoneally (i.p).

### Collection of plant material

The leaves of *M. peregrina *were collected in June 2012 from Wadi Bin-Hammad Valley (Karak, south of Jordan). Professor Sawsan Oran, plant taxonomist at the University of Jordan, authenticated the leaves.

### Preparation of plant extract

The dried leaves of *M. peregrina* were extracted by maceration in 96% methanol (*Scharlau Chemie*, Spain). Rotary evaporator was used to evaporate methanol under reduced pressure and at a temperature not more than 45^o^C. The extract was stored at -20°C, and it was freshly prepared, before its use, by dissolving it in the sterile normal saline solution.

### Experimental animals

All employed procedures in this study complied with the guidelines of the International Association for the Study of Pain (17) and were approved by the ethical committee at Al-Ahliyya Amman University (ethical approval no. AAU-2/4/2018). Male BALB/c mice (weight: 20-25 g) were kept at standard laboratory conditions (at 23±2^o^C in both dark and light environments consecutively). Food and water were provided ad libitum. The animals were allowed to acclimatize to the experimental laboratory conditions for 120 minutes before the commencement of experiments.

### Pretreatment of animals

In all the experiments, animals were pretreated i.p with 10 mL/kg vehicle (sterile normal saline solution, control) along with 200 mg/kg or 400 mg/kg *M. peregrina *methanolic extract 30 minutes before the commencement of the experiments. Diclofenac sodium (30 mg/kg) was used as a standard drug similar to the research of Jaffal and Abbas (18). Methysergide (5 mg/kg) or yohimbine (1 mg/kg) was injected 15 minutes before the administration of a fixed dose of *M. peregrina *extract (200 mg/kg or 400 mg/kg). The choice of doses of antagonists was based on the previously published studies (19,20).

### Writhing test

Writhing test was conducted, as per Koster method (21), via the administration of acetic acid solution (1%, 10 mL/kg) i.p in the animals 30 minutes after receiving the vehicle solution, *M. peregrina *extract (200 mg/kg or 400 mg/kg), or diclofenac sodium. Each group consisted of eight animals (mice). After 10 minutes of acetic acid administration, the number of writhes was counted for 20 minutes. A writhe is considered as a contraction of the abdominal muscles accompanied by the elongation of the body and extension of the forelimbs. The percentage inhibition of abdominal cramps was calculated by using the following formula:

% inhibition = Average number of writhes in control - Average number of writhes in treated animals × 100%

Average number of writhes in control.

### Paw-licking test

Paw-licking test was performed after the intraplantar administration of 2.5% formalin (20 µL) to the left hind paw of the mouse. The total time spent in licking the injected paw, lifting the leg, or exhibiting a flinching behavior were recorded in the first 5 minutes after the administration of formalin (early phase) and during the late phase (25-30 minutes after injection). The percentage inhibition was calculated according to the following formula:

% inhibition= Average time of licking in control - average time of licking in treated animals × 100%

Average time of licking in control

### Hot plate test

The hot plate test was used to determine latencies in pain reaction. Mice reactions were assessed by individually placing the animals into a transparent container on a hot plate at 55±1^o^C. Each mouse underwent this procedure only once. The time between the animal’s placement and first jump was recorded as a measure for the latency of pain reaction. A cut-off time of 60 seconds was determined to avoid tissue damage.

### Tail-immersion test

Tail-immersion test was performed by dipping the tail in water at 55±1^o^C. The time starting from immersing the tail in water till the appearance of the first flick was calculated. A cut-off time of 10 seconds was determined.

### Liquid chromatography-mass spectrometry (LC-MS)

LC-MS separation was performed in the mobile phase. This phase have solvents A and B in gradient, in which A contained 0.1% (v/v) formic acid in water and B had 0.1% (v/v) formic acid in acetonitrile. Agilent ZORBAX Eclipse XDB-C18 column (2.1×150 mm ×3.5 μm) was used in this procedure. The oven was set at 25 °C, and the volume of injection was 1 μL containing 18 mg/mL in methanol. We used Shimadzu LC-MS 8030 with electrospray ion mass spectrometer (ESI-MS) to monitor the eluent under positive ion mode. Then, we scanned it from 100 to 1,000 mass/number of ions (m/z). ESI was performed by using skimmer 65 V and at a fragmentor voltage of 125 V. Highly pure (99.99%) nitrogen was used as drying gas at 10 L/min flow rate, capillary temperature at 350 °C, and nebulizer at 45 psi. The sample was injected to the mass detector by using the LC-30AD pump, Shimadzu CBM-20A system controller, cooler, and CTO-30 column oven with the SIL-30AC autosampler. The results were validated by running the authentic standard compounds and referring to the literature as in the research of (16).

### Protein preparation and homology modeling

The homology model for α_2_-adrenergic receptor was developed from the SWISS-MODEL server (22). Other software included in this study were Discovery Studio Visualizer 4.0 (Accelrys Software Inc, San Diego; http://www.accelrys.com), ACD/ChemSketch, (www.acdlabs.com), and AutoDock4 (23).

The protein sequence of α_2_-adrenergic receptor was selected from the universal protein source under the code no. P08913. The homology model of the protein was built at the SWISS-MODEL web server program. Initially, BLAST (24) was used to search for the target sequences against the primary amino acid sequence. Thereafter, the quality of the template was predicted from the target-template alignment features. Further, the best-quality templates were chosen for building the model.

### Molecular Docking

ACD/ChemSketch software was used to construct the chemical structures of major compounds in *M. peregrina* extract. These compounds were chrysoeriol 7-O-diglucoside, lupeol acetate, quercetin, and rutin. The compounds were drawn and saved as MOL files by ChemSketch software and then converted to PDB files. Ligand files in the PDB format were prepared by AutoDockTools. Each compound was opened separately, charges were added, and all the hydrogen atoms were merged. Molecular docking simulations of the compounds were performed by utilizing AutoDock 4.2. Kollman and Gasteiger charges were added to both proteins and plant compounds, respectively. A set of grid maps were created by using AutoGrid 4 (The Scripps Research Institute, San Diego, CA, USA). Then, a grid box was utilized to select the area of the protein structure to be mapped. The box size was set to 22.5, 22.5, and 22.5 Å (x, y, and z, respectively). Energy optimization and minimization was conducted by applying Lamarckian genetic algorithm in the docking simulation (16).

### Statistical analysis

All data of this study passed the normality test (Shapiro-Wilk test). Brown-Forsythe and Welch One-Way analysis of variance tests for parametric analysis were used to examine the statistical difference between the groups. Version 6 of GraphPad Prism was chosen to perform the statistical analysis of this study.

## RESULTS

**Figure 1:**
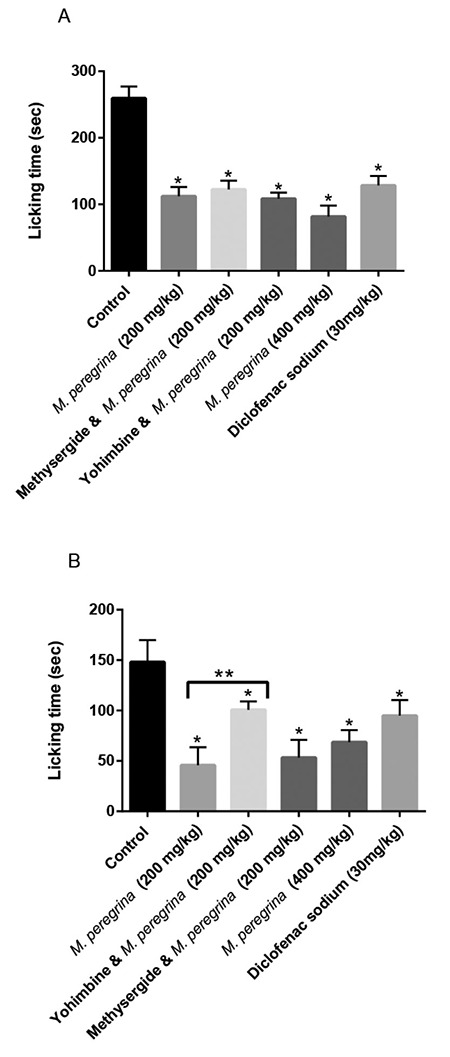
Results of paw-licking test (formalin test). (a) Early phase (0-5 minutes after injection) (b) Late phase (25-30 minutes after injection). *Significant difference from the control (p<0.05), **P<0.05 significantly different from M. peregrina (200 mg/kg)

In the writhing test, 200 mg/kg and 400 mg/kg of *M. peregrina* extract inhibited abdominal cramps by up to 55.97% and 88.00%, respectively, as compared to inhibition percentage of 47.69% produced by 30 mg/kg of diclofenac sodium. Yohimbine antagonized the action of* M. peregrina* in the writhing test (Table 1). Additionally, the methanolic extract of *M. peregrina* reduced the time of paw-licking in the early and late phases of formalin test. high (400 mg/kg) and low (200 mg/kg) doses of *M. peregrina* extract exhibited inhibition percentages of 59.6% and 56.8%, respectively, whereas 30 mg/kg diclofenac sodium produced an inhibition percentage of only 50.5% in the early phase of formalin test (Figure 1A). High (400 mg/kg) and low (200 mg/kg) doses of *M. peregrina* extract exhibited inhibition percentages of 50.1% and 65.5%, respectively, as compared to an inhibition percentage of 48.4% achieved by 30 mg/kg diclofenac sodium in the late phase of formalin test (Figure 1B). This effect (in late phase only) was abrogated by yohimbine, but not by methysergide.

In the hot plate, but not tail-immersion test, a high dose of *M. peregrina* (400 mg/kg) extract increased the latency time after 30 minutes of its administration. Yohimbine antagonized the action of* M. peregrina* in the hot plate test (Table 2)*.* By using LC-MS, 18 compounds were detected in the extract (Table 3). Chrysoeriol 7-O-diglucoside, lupeol acetate, quercetin, and rutin were the major compounds present in this extract.

**Figure 2:**
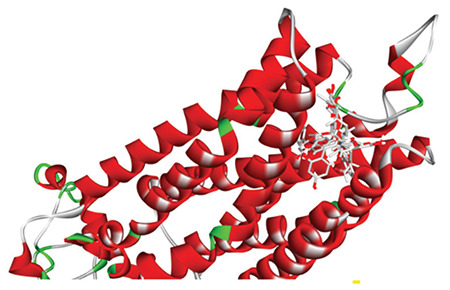
Solid ribbon representation of α_2_-adrenergic receptor docked with the major compounds in *M. peregrina* extract, namely chrysoeriol 7-O-diglucoside, lupeol acetate, quercetin, and rutin, in the active site of the receptor.

SWISS-MODEL had generated around 531 templates, of which the top 50 filtered templates can be found in the supplementary material (Table S1). The homology model of α_2_-adrenergic receptor having a resolution of 1.96 Å was validated by using the Ramachandran plot (Figure S1 in supplementary materials). Quality estimate of the homology model is presented in Figure S2 while predicted local similarity to target for the homology model is shown in Figure S3 in supplementary material. Figure 2 shows all the successfully docked plant constituents against the α_2_-adrenergic receptor model, and Table 4 shows the results of lowest binding energies .

**Figure 3:**
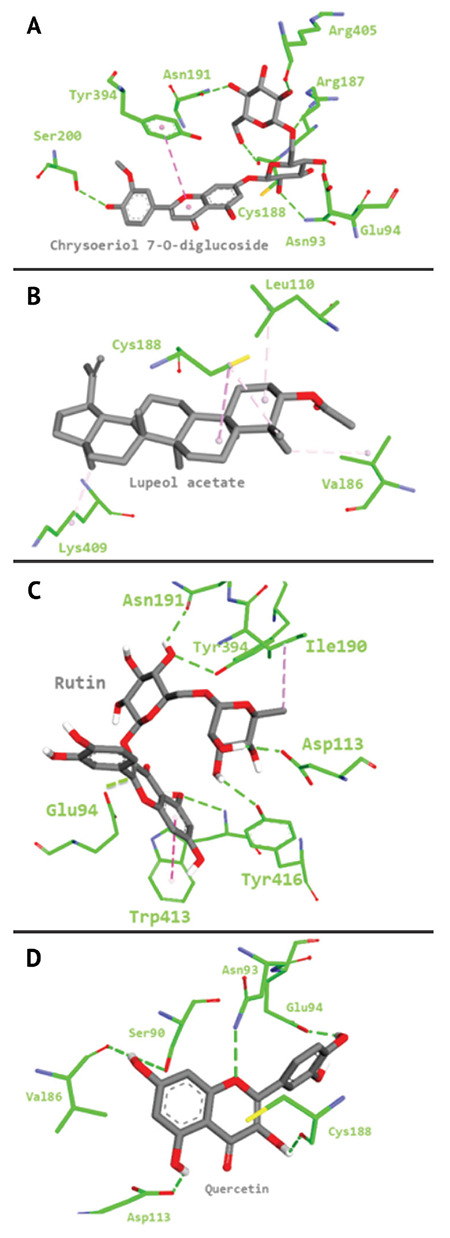
Stick representation of a. chrysoeriol 7-O-diglucoside, b. lupeol acetate, c. rutin, and d. quercetin in the active site that forms hydrophobic (pink dots) interactions and hydrogen bond (green dots) with α_2_-adrenergic receptor.

The docking results showed a higher binding affinity for the compounds, namely chrysoeriol 7-O-diglucoside, quercetin, and rutin, with a low affinity for lupeol acetate. Further investigation of these compounds in the binding site have revealed that chrysoeriol 7-O-diglucoside, quercetin, and rutin participate in the hydrogen bond interactions as well as hydrophobic interactions within the binding site, whereas lupeol acetate participates only in the hydrophobic interactions (Figure 3).

## DISCUSSION

In our study, *M. peregrina* exhibited marked analgesic properties induced by acetic acid in the writhing test. The percentage inhibition produced by 200 mg/kg  and 400 mg/kg doses were 55.97% and 88%, resprctively. Similar findings were obtained by Elbatran et al. (12) in which 113.4 mg/kg of *M. peregrina* decreased acetic acid-induced abdominal cramps by 70.7% in mice. Additionally, at the doses of 100 and 200 mg/kg,* M. oleifera* extract significantly decreased writhes (25). This effect was not reversed by naloxone, thereby indicating a peripheral non-opioid mechanism of action (26). Bhattacharya et al. (27) also found that *M. oleifera* extract exhibited analgesic activity at 100, 200, and 400 mg/kg in acetic acid-induced abdominal cramps in the writhing test, further showing percentage inhibitions of 32.21%, 59.71%, and 78.61% of writhes, respectively, as compared with the control group (27).

*M. peregrina* leaf extract demonstrated noticeable antinociception in the early and late phases of the formalin test. The administration of formalin causes three distinct periods with high licking behavior: an early phase lasting the first 5 minutes, and a late phase in the last 5 minutes after the injection. The early phase (neurogenic pain) is due to the direct activation of the primary afferent fibers. The late phase is known as inflammatory pain, thereby representing the effect in the primary afferents and central sensitization of spinal cord circuits secondary to the events that occurred during Phase I (28)*. *The lowest dose of *M. peregrina* (200 mg/kg) was more active in the late phase than in the early phase and was more active than the higher dose (400 mg/kg). This can be explained by the non-specific effect of compounds in a higher dose with receptors that can block the interaction of active compounds with the receptor involved in the antinociceptive action. As per our knowledge, this study is the first one to report on the activity of *M. peregrina* in the formalin test. Similarly,* M. oleifera *leaves exhibited antinociceptive activity in the late phase (25,26). Both polar and non-polar extracts of *M. oleifera* leaves were effective in both phases of formalin test. Due to the presence of central and peripheral actions of non-polar active compounds, the hexane extract was more effective than the ethanolic extract (29).

In the hot plate test, a high dose of *M. peregrina* (400 mg/kg) extract increased the latency time after 30 minutes of its administration. Similar results were reported by Elbatran et al. (12). In search for the mechanism of antinociceptive action of* M. peregrine*, the antagonists for adrenergic and 5-hydroxytryptamine type 2 (5-HT2) serotonergic receptors were used in this study since previous studies suggested that the closely related species of* M. oleifera *interact with both dopaminergic and 5-HT2 serotonergic receptors (30). This study showed that yohimbine antagonized the action of* M. peregrina* in the hot plate test. As per our knowledge, this is the first report that shows the involvement of α_2_-adrenergic receptors in the action of *M. peregrina*. The related species of *M. oleifera* showed significant analgesic dose-dependent activity in the hot plate test (27). Study suggested that the effect of *M. oleifera* in the hot plate test is modulated at the central antinociceptive level via opioid receptors since the use of naloxone (5 mg/kg) reversed the effect of extract (26).

In the tail-immersion test, *M. peregrina* extract had no effect on the latency time of tail immersion after the administration of extract. In contrast, *M. oleifera* produced considerable antinociceptive action by enhancing tail-immersion latency period at 30 minutes (31). This could be explained by the presence of different phytoconstituents in the two species.

Both tail-immersion and hot plate tests are thermal acute pain tests. However, tail-immersion test works at the spinal level, whereas the hot plate test is a supraspinally controlled test (32). To the best of our knowledge, this study is the first one to report the lack of spinal reflexive action of *M. peregrina*.

Elbatran et al. (12) isolated four flavonoids from the aerial parts of *M. peregrina *and found that the major flavonoids are quercetin-3-0*-*rutinoside (rutin), quercetin, chrysoeriol-7*-*0-rhamnoside, and 6,8,3,5-tetramethoxy apigenin. These flavonoids showed both analgesic and anti-inflammatory properties. Additionally, the aerial sections of *M. peregrina* have β-sitosterol-3-O-glucoside, β-sitosterol, α-amyrin, β-amyrin, lupeol acetate, apigenin, 6-methoxy-acacetin-8-C-β-glucoside, neochlorogenic acid, rhamnetin, and rhamnetin-3-O-rutinoside (13). Lupeol acetate isolated from *M. peregrine* has a well-documented analgesic activity (33).

Depending on the previous results, we propose that the plant extract will interact with α_2_-adrenergic receptor to exert its analgesic activity. Thus, it will be useful to determine the active compounds that are responsible for this action. Due to the absence of complete α_2_-adrenergic receptor crystal structure, homology modeling is considered as an attractive technique for obtaining the structure. Such technique has proven to be an appropriate choice to obtain the useful 3D structure of the protein (34). Furthermore, molecular docking simulations have shown a good affinity of chrysoeriol 7-O-diglucoside, quercetin, and rutin toward the active site of the receptor.

In conclusion, our results suggest an interaction with α_2_-adrenergic receptor as a possible mechanism of analgesic action of* M. peregrina*. Depending on the molecular docking results, the activity of *M. peregrina* extract with α_2_-adrenergic receptor could be due to the binding of chrysoeriol 7-O-diglucoside, quercetin, and rutin compounds to the receptor.

## Figures and Tables

**Table 1:**
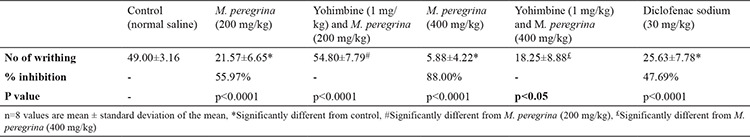
Writhing test results

**Table 2:**
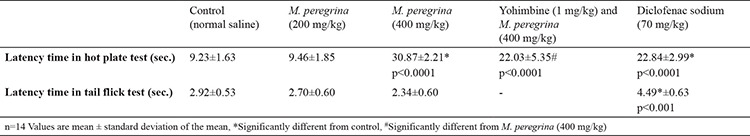
Results of thermal antinociceptive tests: hot plate and tail flick tests

**Table 3:**
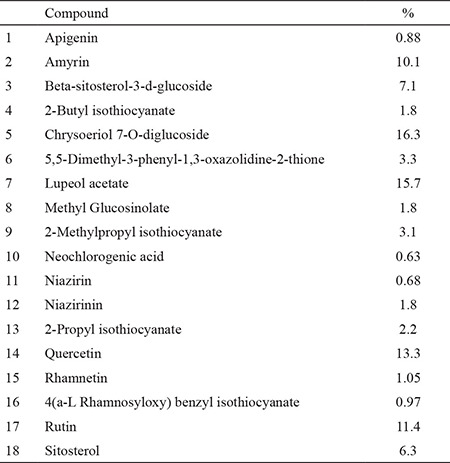
Chemical constituents of *M. peregrina* as detected by liquid chromatography-mass spectrometry

**Table 4:**
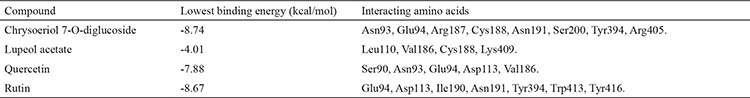
The lowest binding energies obtained from AutoDock 4.2 for plant constituents against α_2_-adrenergic receptor and the interacting amino acids.
